# Vitamin D Status and Its Relationship with Metabolic Markers in Persons with Obesity and Type 2 Diabetes in the UAE: A Cross-Sectional Study

**DOI:** 10.1155/2014/869307

**Published:** 2014-10-13

**Authors:** Amena Sadiya, Solafa M. Ahmed, Sijomol Skaria, Salah Abusnana

**Affiliations:** Rashid Center for Diabetes and Research, Ministry of Health, Ajman, UAE

## Abstract

*Aim.* To report vitamin D status and its impact on metabolic parameters in people in the United Arab Emirates with obesity and type 2 diabetes (T2D). *Methodology.* This cross-sectional study included 309 individuals with obesity and T2D who were randomly selected based on study criteria. Serum concentrations of 25-hydroxy vitamin D (s-25(OH)D), calcium, phosphorus, parathyroid hormone, alkaline phosphatase, glycemic profile, and cardiometabolic parameters were assessed in fasting blood samples, and anthropometric measurements were recorded. *Results.* Vitamin D deficiency (s-25(OH)D < 50 nmol/L) was observed in 83.2% of the participants, with a mean s-25(OH)D of 33.8 ± 20.3 nmol/L. Serum 25(OH)D correlated negatively (*P* < 0.01) with body mass index, fat mass, waist circumference, parathyroid hormone, alkaline phosphatase, triglycerides, LDL-cholesterol, and apolipoprotein B and positively (*P* < 0.01) with age and calcium concentration. Waist circumference was the main predictor of s-25(OH)D status. There was no significant association between serum 25(OH)D and glycemic profile. *Conclusion.* There is an overwhelming prevalence of vitamin D deficiency in our sample of the Emirati population with obesity and T2D. Association of s-25(OH)D with body mass index, waist circumference, fat mass, markers of calcium homeostasis and cardiometabolic parameters suggests a role of vitamin D in the development of cardiometabolic disease-related process.

## 1. Introduction

Vitamin D is a fat-soluble vitamin with hormonal functions, and concentrations of serum 25-hydroxyvitamin D (s-25(OH)D) are largely determined by ultraviolet light exposure, dietary intake, and supplementation [1]. Vitamin D has traditionally been associated with calcium metabolism and bone mineralization. However, recent evidence from various research reports has suggested a role in the progression of chronic diseases such as diabetes mellitus and cardiovascular diseases but with inconsistent results [2, 3]. Vitamin D deficiency is an important public health problem worldwide [4], and although the Middle East receives ample sunshine, people living in this region (15° to 36°N) have high prevalence of hypovitaminosis D across all age groups [5, 6]. This has been attributed to several factors including limited exposure to sunlight, low dietary intake of vitamin D_2_, dark skin color, clothing, and religious practices that restrict sunlight exposure [6, 7]. These conditions may vary between countries based on geographical location and ethnicity.

The high prevalence of diabetes and obesity in the United Arab Emirates (UAE) has motivated concern about hypovitaminosis D, since several studies have reported increased vitamin D deficiency in persons with obesity and type 2 diabetes mellitus (T2D) compared to healthy controls [5, 8]. Most studies have focused on vitamin D status and the incidence of diabetes, while few have evaluated the relationships between vitamin D status and various pathophysiological and metabolic parameters in patients with T2D [9]. Although some epidemiological studies have shown s-25(OH)D to be associated with insulin sensitivity and glucose tolerance [10], the relationships between vitamin D status and metabolic parameters in various ethnic groups are not well known, a situation that leaves ample scope for further investigation into the relationship between vitamin D status and clinical variables in patients with obesity and T2D.

Information about vitamin D status in adults in the Middle East with obesity and T2D is scarce. Furthermore, the relationship between hypovitaminosis D and different metabolic parameters has not been studied in depth. To our knowledge no studies have been published about the population residing in the UAE with obesity and T2D. The present study was designed to determine vitamin D status and search for possible associations between serum vitamin D concentration and metabolic parameters in persons with obesity and T2D in this country.

## 2. Methods

### 2.1. Participants

This cross-sectional study was done at Rashid Center for Diabetes and Research, a tertiary diabetes care facility in Ajman, UAE. The UAE is situated in the northern hemisphere between 22° and 26.5° north of the equator. A total of 2101 patients registered from December 2010 to December 2013 were reviewed based on the inclusion and exclusion criteria, and 309 men and women were randomly selected for data analysis. The inclusion criteria were UAE nationality, age between 30 and 60, diagnosis of T2D, and body mass index (BMI) ≥ 30 kg/m^2^. Participants were excluded if they were diagnosed with type 1 diabetes and had a history or evidence of parathyroid or calcium related diseases, history or evidence of endocrine diseases including hyperthyroidism, hypothyroidism, adrenal disease, and pituitary disease, or history of major renal, liver, heart, blood, or neurological disease, as judged by the investigating physicians. All patients gave their written informed consent acknowledging the use of their data for the study as approved by the Regional Ethics committee of the Ministry of Health, UAE. The study was conducted in accordance with the Declaration of Helsinki.

### 2.2. Methods

All patients were examined by a physician at their first visit to the center, and demographic data, medical history, presence of comorbidities, and medications used were recorded as part of standard clinical practice in the health care information system. A standard pretested questionnaire was completed by a subgroup of 154 participants assisted by a study staff member, in order to record information about outdoor exposure to sunlight, use of sunscreens, clothing, skin color (Fitzpatrick scale), and intake of food sources rich in dietary vitamin D_2_. Anthropometric measurements (height, weight, waist circumference (WC), and body composition) were also recorded. Body weight and height were measured with an electronic balance and stadiometer (Seca, Hamburg, Germany, capacity: max 250 kg, range: 110 cm/43 ins to 200 cm/79 ins) and recorded to the nearest 0.1 kg or 0.1 cm. Body composition was determined by bioelectric impedance with an InBody-230 analyzer (Biospace, Dogok-dong, South Korea). Body mass index (weight (kg)/height^2^ (m^2^)) was also calculated. Waist circumference was defined as the abdominal circumference immediately above the iliac crests. Systolic and diastolic blood pressure were measured in the right arm while the participant was seated and after he or she had rested for at least 10 min. A fasting (12 hours) venous blood sample (8 mL) was collected and used to determine glycemic profile (fasting blood glucose (FBG), HbA1c, and C-peptide), cardiometabolic parameters (serum cholesterol, triglycerides (TG), high-density lipoprotein cholesterol (HDL-C), low-density lipoprotein cholesterol (LDL-C), apolipoprotein A (Apo A), apolipoprotein B (Apo B), C-reactive protein (CRP)), markers of calcium homeostasis (serum 25(OH)D, calcium, phosphorus, parathyroid hormone (PTH), and alkaline phosphatase (ALP)), and serum creatinine. All values were measured with a Roche COBAS 6000 analyzer (Mannheim, Germany). Serum 25(OH)D was measured with an immunochemiluminescence method in a DiaSorin Liaison analyzer (Saluggia, Italy). The analyzing laboratory participated in the vitamin D external quality assessment scheme (DEQAS). All parameters were measured at the same time or close to the date of s-25(OH)D determination.

Vitamin D status was recorded based on serum concentration of 25(OH)D as severely deficient (<25 nmol/L), deficient (<50 nmol/L), insufficient (<75 nmol/L), or normal (>75 nmol/L) [5, 6].

### 2.3. Statistical Analysis

Normal distribution of all the data sets was confirmed by histogram plots, and the results were presented as the mean ± standard deviation. Pearson's correlation coefficient was calculated to identify bivariate associations between s-25(OH)D and other covariates. Additionally, the participants were classified into subgroups based on gender, medication use, glycemic profile, and fat mass to determine the strength of the relationships of metabolic markers with s-25(OH)D. We also stratified our sample into subgroups based on s-25(OH)D status for comparison with one-way ANOVA, and the Bonferroni method was used for post hoc comparisons. To evaluate predictors of 25(OH)D status we used regression analyses with age, BMI, WC, fat mass, LDL-C, total cholesterol, and TG. The criterion for statistical significance (two-tailed tests) was *P* ≤ 0.05.

## 3. Results

Table 1 shows the participants' responses to the questionnaire on factors affecting vitamin D status, that is, lifestyle, dietary intake, and vitamin D supplementation. Seventy-nine percent of the participants had a skin score of 4 and 5, that is, brown to dark brown skin characterized by easy tanning. The dietary intake of foods rich in vitamin D_2_ and vitamin D_3_ supplementation are also shown. Sixty-eight percent of the participants were on oral antidiabetic drugs only, whereas the rest were on both insulin and oral antidiabetic drugs. The mean duration of T2D since diagnosis was 12.0 ± 7.5 years.

**Figure 1:**
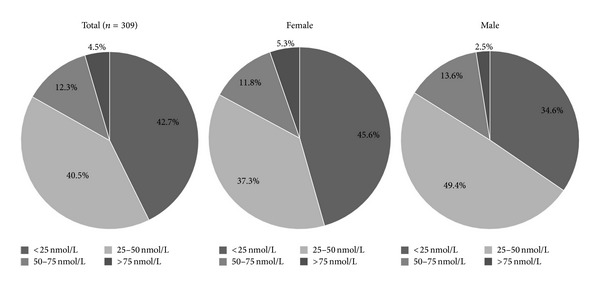
Serum 25(OH) vitamin D status in males and females according to cut-off values. Note: vitamin D deficiency was defined as s-25(OH)D < 50 nmol/L.

Figure 1 illustrates s-25(OH)D status in participants with obesity and T2D and the distribution in men and women of the categories of vitamin D status according to cut-off values. The overall serum vitamin D status was deficient (<50 nmol/L) in 83.2% of the participants, and only 4.5% of them had a normal s-25(OH)D status (>75 nmol/L). The prevalence of severe 25(OH)D deficiency (<25 nmol/L) was higher in women than in men (*P* < 0.05).

The clinical characteristics of our participants and the associations of s-25(OH)D with different metabolic parameters are shown in Table 2. The mean levels of FBG, HbA1c, LDL-C, and CRP were higher than the recommended target, and HDL-C levels were lower (ADA guidelines). Serum 25(OH)D was positively associated with age and negatively associated with weight, BMI, WC, and fat mass, whereas no association was evident with lean body mass. In addition, s-25(OH)D was positively associated with calcium and inversely related to PTH and ALP (*P* < 0.01). The lipid profile and Apo B level were associated with s-25(OH)D, but no significant association was observed between s-25(OH)D and blood pressure, glycemic profile, CRP, creatinine, or Apo A.

We divided the participants into subgroups based on glycemic control (HbA1c < 7%, 7% to 9%, and >9%), gender, duration of diabetes (<5, 5 to 10, 10 to 15, >15 years), and medication use (oral antidiabetics only versus insulin therapy plus oral antidiabetics) to search for further associations between these variables and vitamin D status. However, there were no significant differences between the quartiles, and no associations were found with glycemic profile (data not shown).

The association between s-25(OH)D and HDL-C, LDL-C, total cholesterol, and TG was stronger in males (*r* = 0.21, −0.36, −0.33, and 0.29, resp.) than in females (*r* = 0.12, −0.09, −0.09, and −0.11, resp.), whereas the association with Apo B was stronger in females (*r* = −0.21 in women versus −0.06 in men). In addition, we found a significant association between s-25(OH)D and LDL-C (*r* = −0.21), cholesterol (*r* = −0.22), and TG (*r* = −0.18) (*P* < 0.01) in the subgroup of patients on oral antidiabetic medication only.

Table 3 compares the results for different metabolic variables across three categories of vitamin D status according to cut-off values for s-25(OH)D. There were no significant differences between these subgroups in any of the variables in the glycemic profile. The values for the cardiometabolic variables, that is, LDL-C, cholesterol, TG, Apo B, and CRP, decreased steadily in subgroup with 25-nmol/L higher s-25(OH)D. As s-25(OH)D status improved, calcium levels increased, whereas PTH and ALP levels decreased. Linear regression analysis identified PTH (*β* = −1.67, *P* < 0.001), calcium (*β* = 45.6, *P* < 0.001), and ALP (*β* = −0.21, *P* < 0.001) as factors associated with serum vitamin D status.

## 4. Discussion

The main finding in this cross-sectional study is the very high prevalence (83.2%) of vitamin D deficiency (<50 nmol/L), with a mean s-25(OH)D of 33.8 ± 20.3 nmol/L in Emirati patients with obesity and T2D. This was similar to the mean s-25(OH)D value reported for patients in Saudi Arabia with diabetes and obesity (35.5 ± 30.6 nmol/L, 39.18 ± 18.72 nmol/L) [5, 11]. However, our observed mean was far lower than the value reported for Japanese patients with diabetes (42 ± 17.7 nmol/L) [12] and a sample of patients in the USA (57.15 nmol/L) [13]. The discrepancy between our results and the findings in other ethnic groups may reflect ethnic differences, lifestyle factors, and differences in the dietary intake of fortified foods. Our results confirm and augment the body of evidence that vitamin D deficiency is alarmingly high among people in the UAE with diabetes and obesity. In the present study there was no significant difference in vitamin D status between males and females (35.1 ± 19.2 nmol/L versus 33.3 ± 20.7 nmol/L), a finding consistent with an earlier study of people in Saudi Arabia with diabetes and obesity [11]. Nevertheless, severe vitamin D deficiency (<25 nmol/L) was more frequent in females than the males (45.6% versus 37.3%). The higher percentage of severe deficiency may be attributable to differences between study populations in lifestyle factors, cultural practices, and fat mass [14, 15].

Vitamin D is a nutrient that is derived from dietary sources, and up to 80% is produced by synthesis in the skin under the influence of ultraviolet-B radiation from sunlight. In the present study the low levels of s-25(OH)D may be a result of restricted exposure to direct sunlight, insufficient vitamin D intake, or both. Forty-two percent of our participants were exposed to direct sunlight for less than 5 min/day, and they often avoided exposure in the afternoons due to high temperatures. About one-fourth (24.2%) wore a sunscreen before going outdoors, and all participants wore their traditional attire (an* abaya* for females and a* kandoora* for males) outdoors, which reduced the surface area exposed to direct sunlight [16]. The black clothing (*abaya*) worn by female participants blocks 100% of ultraviolet-B radiation [17]. Moreover, darker skin produces less vitamin D than lighter shades of skin [18], and 79% of our participants had brown to dark brown skin. Although the consumption of milk, yoghurt, and cheese was fairly high with 64% consuming these items 1 or 2 times per day, it is not known whether milk sold in the UAE is adequately fortified with vitamin D. Studies from the USA and Canada show that fortified milk did not contain the amount of vitamin D claimed on the label [19]. Our participants consumed considerable amounts of fish but not the oily sea fishes that are rich in vitamin D_3_. Hence, we assume that the frequency of consumption of foods rich in vitamin D_2_ may be insufficient to contribute to the daily recommended dietary allowance of 600 IU/day [20]. Some of our participants also reported consuming cod liver oil (17.6%) and vitamin D supplements (24.2%), but the average dose was 400 IU vitamin D_3_/day.

Our results indicate a positive relationship between s-25(OH)D and age, as supported by similar evidence in the Middle East region [5, 6]. This relationship may be partly elicited by multivitamin supplementation in elderly patients in clinical practice, which may have contributed to increased s-25(OH)D in this age group.

It is well established that obesity is associated with vitamin D deficiency, and our data confirm previous reports of a negative association between s-25(OH)D and BMI, fat mass, and WC [15, 21]. Low levels of s-25(OH)D have been attributed to sequestration of vitamin D by the adipose tissue [22], dilution of ingested or cutaneously synthesized vitamin D in the enlarged fat mass [23], reduced release of vitamin D into the systemic circulation, and low exposure to direct sunlight due to reduced physical activity [15, 24]. Serum levels of 25(OH)D correlated negatively with WC after adjustment for confounders such as BMI, fat mass, and age. As suggested by Cheng et al. in 2010 [15], both subcutaneous adiposity and visceral adiposity are associated with low vitamin D concentrations. The vitamin D system is known to be altered in persons with obesity, and this may have implications for the development of both obesity itself and its comorbidities.

Our study adds to the evidence that serum vitamin D status is not associated with the glycemic profile in Emiratis with obesity and T2D. However, the association of serum 25(OH)D with C-peptide (*r* = −0.17) and HOMA-IR (*r* = −0.16) was significant (*P* < 0.01) in the subgroup of participants who were using oral medications, whereas no association was observed with duration of T2D. Previous studies have reported inconclusive results regarding the association between vitamin D status and HbA1c [5, 8, 25], and the lack of a clear trend among different studies suggests that ethnicity may have an effect on this relationship. However, a randomized clinical trial to study the effect of vitamin D_3_ supplementation in patients with T2D found no effect on glycemic profile [9].

Our results confirmed the association between 25(OH)D, PTH, and calcium that has been described in the literature [1]. Our findings also indicate that there are a negative relationship between s-25(OH)D and LDL-C, cholesterol, TG, and Apo B and a positive relationship with HDL-C. Similar associations with lipid profile parameters were found in 108 patients from Iran with T2D [26]. Moreover, vitamin D was inversely related to atherogenic dyslipidemia in some studies but not in others. A review of 22 cross-sectional studies showed a positive association between vitamin D and HDL-C resulting in a favorable ratio of LDL-C (or cholesterol) to HDL-C [27–29]. There was also consensus between studies on the negative relationship between vitamin D and TG [30]. Although there is no general agreement among interventional studies regarding the effects of 25(OH)D on lipid levels, it has been suggested that vitamin D can modify the lipid profile via both direct and indirect effects. Vitamin D may decrease serum TG through a regulatory action that increases the activity of lipoprotein lipase in persons with obesity [31]. In vitro studies have found that PTH decreases lipolysis (by increasing the level of cystolic calcium) and increases the expression of fatty acid synthesis [32, 33]. Calcium influences lipid levels by interfering with fatty acid absorption via the formation of insoluble calcium—fatty complexes in the gut; this results in an increase in the conversion of cholesterol to bile acids [34]. A strong association between s-25(OH)D and Apo B was also observed in our study, as in an earlier report in normal adults [35]. The association of s-25(OH)D with cardiometabolic parameters in our study population also appeared to be influenced by other factors such as the duration of diabetes (12.0 ± 7.5 years) and BMI (36.9 ± 6.0 kg/m^2^).

Two particular strengths of our study are that we selected a fairly large sample and included individuals who comprised a homogenous Emirati population of people with obesity and T2D who share a similar lifestyle. A potential limitation of this study was that the information on dietary intake was obtained from self-reports, which should be considered with due caution. Our findings must accordingly be interpreted in the light of these acknowledged limitations.

## 5. Conclusion

The main finding of this cross-sectional study is the very high prevalence of vitamin D deficiency in our sample of the Emirati population with obesity and T2D. Serum concentrations of 25(OH)D were not associated with any particular characteristics of the glycemic profile but were strongly associated with BMI, WC, and fat mass, markers of calcium homeostasis and cardiometabolic parameter. Although our study design was cross-sectional and no inferences should be drawn regarding causality effects, our findings, together with previous research in other settings, suggest that an adequate vitamin D status may help prevent the development of cardiometabolic disease-related processes. However randomized controlled trials are suggestive to prove the causality in this area.

## Figures and Tables

**Table 1:** Participants' responses to questionnaire on lifestyle, dietary intake, and supplemental vitamin D.

Variable	Percent (%)
Fitzpatrick skin score grades 4 and 5	79
Using sunscreen	24.2
Exposure to direct sunlight <15 min/day	71
Milk and milk product (servings/day)	
None	23
1 to 2	64
3 to 4	9
>4	4
Fortified breakfast cereal (servings/week)	
None	73.6
1 to 2	12.8
3 to 4	12
>4	1.6
Fish (servings/week)	
None	23
1 to 2	46.2
3 to 4	25.3
>4	5.5
Cod liver oil	17.6
Vitamin D supplement	24.2

**Table 2:** Clinical characteristics of participants and correlation with serum 25(OH)D.

Parameters	Mean ± SD	*r*	*P* value
*N* (males)	309 (81)		
Age (years)	48.7 ± 7.8	0.26	0.00
Weight (kg)	94.0 ± 17.1	−0.13	0.02
Body mass index (kg/m^2^)	36.9 ± 6.0	−0.15	0.01
Waist circumference (cm)	111 ± 12	−0.17	0.00
Fat mass (kg)	43.0 ± 11.1	−0.16	0.01
Systolic blood pressure (mmHg)	133 ± 17	0.07	0.22
Diastolic blood pressure (mm Hg)	72 ± 10	−0.01	0.86
25(OH) vitamin D (nmol/L)	33.8 ± 20.3	1	n/a
Calcium (mmol/L)	2.31 ± 0.1	0.21	0.00
Phosphorus (mmol/L)	1.12 ± 0.17	0.12	0.04
Parathyroid hormone (pmol/L)	5.7 ± 3.7	−0.28	0.00
Alkaline phosphatase (IU/L)	84.5 ± 22.8	−0.16	0.00
Fasting blood glucose (mmol/L)	10.5 ± 4	0.04	0.48
HbA1c (%)	9.0 ± 2.0	0.01	0.86
C-peptide (nmol/L)	1.14 ± 0.52	−0.07	0.23
Hs-C-reactive protein (mg/L)	9.5 ± 9.9	−0.09	0.12
Creatinine (mmol/L)	56.1 ± 20.3	0.07	0.25
HDL-cholesterol (mmol/L)	1.1 ± 0.3	0.12	0.03
LDL-cholesterol (mmol/L)	2.8 ± 0.9	−0.16	0.00
Total cholesterol (mmol/L)	4.6 ± 1.1	−0.16	0.00
Triglycerides (mmol/L)	1.7 ± 0.8	−0.15	0.01
Apolipoprotein A	1.36 ± 0.22	0.07	0.30
Apolipoprotein B	0.88 ± 0.26	−0.19	0.00

Data are presented as the mean ± SD. *r* is the correlation coefficient for each variable versus serum concentration of s-25(OH)D.

**Table 3:** Demographic, anthropometric, and laboratory variables in subgroups with different serum 25(OH) vitamin D cut-off values.

Variable	Severely deficient (<25 nmol/L) Group A	Deficient (25–50 nmol/L) Group B	Insufficient (50–75 nmol/L) Group C	*P* value∗
*N* (males)	132 (28)	125 (40)	38 (11)	
Age (years)	46.4 ± 8.0	49.8 ± 7.7	52 ± 5.5^a^	0.00
Weight (kg)	95.4 ± 17.7	94.7 ± 17.1	88.4 ± 13.0^a^	0.03
Body mass index (kg/m^2^)	37.8 ± 6.1	36.8 ± 6.4	34.9 ± 4.5^a^	0.03
Waist circumference (cm)	113 ± 13	111 ± 12	108 ± 9	0.09
Fat mass (kg)	44.9 ± 11.9	42.4 ± 10.7	39.9 ± 9.3^a^	0.03
25(OH) vitamin D (nmol/L)	17.5 ± 4.8	36.2 ± 6.8^a^	59.9 ± 7.3^ab^	0.00
Calcium (mmol/L)	2.29 ± 0.12	2.32 ± 0.09	2.34 ± 0.10^a^	0.02
Phosphorus (mmol/L)	1.09 ± 0.17	1.13 ± 0.17	1.14 ± 0.15	0.18
Parathyroid hormone (pmol/L)	6.6 ± 4.3	5.4 ± 3.3	4.5 ± 2.1^a^	0.01
Alkaline phosphatase (IU/L)	89.4 ± 24.1	81.8 ± 21.1^a^	79.4 ± 20.3^a^	0.01
Fasting blood glucose (mmol/L)	10.6 ± 4.2	10.0 ± 3.7	11.1 ± 4.3	0.28
HbA1c (%)	9.2 ± 2.1	8.7 ± 1.9^a^	9.7 ± 2.2	0.02
C-peptide (nmol/L)	1.1 ± 0.6	1.2 ± 0.5	1.0 ± 0.4	0.20
Hs-C-reactive protein (mg/L)	10.7 ± 10.3	8.8 ± 8.6	6.8 ± 5.3	0.04
HDL-cholesterol (mmol/L)	1.1 ± 0.3	1.2 ± 0.2	1.2 ± 0.3	0.16
LDL-cholesterol (mmol/L)	3.0 ± 1.0	2.9 ± 0.9	2.4 ± 0.7^a^	0.00
Total cholesterol (mmol/L)	4.8 ± 1.1	4.6 ± 1.0	4.1 ± 0.9^a^	0.00
Triglycerides (mmol/L)	1.9 ± 1.0	1.6 ± 0.6^a^	1.5 ± 0.6^a^	0.00
Apolipoprotein B	0.94 ± 0.3	0.86 ± 0.2	0.80 ± 0.2	0.01

Data are presented as the mean ± SD. *P value for differences between groups according to ANOVA. ^a^Significance compared to group A. ^b^Significance compared to group B.
